# Concomitant Deep Vein Thrombosis in Cancer Patients with Unsuspected Pulmonary Embolism

**DOI:** 10.3390/cancers14184510

**Published:** 2022-09-17

**Authors:** Aiham Qdaisat, Adriana H. Wechsler, Maria T. Cruz Carreras, Jazmin R. Menendez, Demis Lipe, Emily A. Highsmith, Mona Kamal, Aisha Al-Breiki, Cristhiam M. Rojas Hernandez, Carol C. Wu, Sai-Ching J. Yeung

**Affiliations:** 1 Department of Emergency Medicine, The University of Texas MD Anderson Cancer Center, Houston, TX 77030, USA; 2 Department of Pharmacy, The University of Texas MD Anderson Cancer Center, Houston, TX 77030, USA; 3 Department of Symptom Research, The University of Texas MD Anderson Cancer Center, Houston, TX 77030, USA; 4 Department of Emergency Medicine, Sultan Qaboos University Hospital, Al Seeb 121, Oman; 5 Section of Benign Hematology, The University of Texas MD Anderson Cancer Center, Houston, TX 77030, USA; 6 Department of Thoracic Imaging, The University of Texas MD Anderson Cancer Center, Houston, TX 77030, USA

**Keywords:** venous thromboembolism, cancer, emergency, unsuspected, incidental, thrombosis, recurrence, survival, pulmonary embolism, concomitant

## Abstract

**Simple Summary:**

Cancer patients have a significantly higher risk of developing venous thromboembolism during their disease course when compared with the general population. During routine staging or follow-up imaging studies, incidental venous thromboemboli, including incidental pulmonary embolisms, can be identified. Identifying factors associated with incidental or unsuspected venous thromboembolism is important and can improve the management plan. In the current study, we found that 20.9% of patients with unsuspected pulmonary embolisms had concomitant deep vein thrombosis, and most of these patients were asymptomatic. In addition, we found that concomitant deep vein thrombosis increases the odds of venous thrombosis recurrence in cancer patients presenting with unsuspected pulmonary emboli. Therefore, for patients with isolated incidental subsegmental pulmonary embolism and concomitant deep vein thrombosis, initiating anticoagulants if no contraindications exist is recommended. In addition, the presence of concomitant deep vein thrombosis among cancer patients with unsuspected pulmonary embolisms is associated with poor short- and long-term outcomes in these patients.

**Abstract:**

Incidental venous thromboembolism (VTE) is common in cancer patients and identifying factors associated with these events can improve the management plan. We studied the characteristics of concomitant deep vein thrombosis (C-DVT) in cancer patients presenting with unsuspected pulmonary embolism (PE) and the association of C-DVT with VTE recurrence and survival outcomes. Patients presenting to our emergency department with confirmed unsuspected/incidental PE between 1 January 2006 and 1 January 2016, were identified. Radiologic reports were reviewed to confirm the presence or absence of C-DVT. Logistic regression analyses and cox regression modeling were used to determine the effect of C-DVT on VTE recurrence and survival outcomes. Of 904 eligible patients, 189 (20.9%) had C-DVT. Patients with C-DVT had twice the odds of developing VTE recurrence (odds ratio 2.07, 95% confidence interval 1.21–3.48, *p* = 0.007). The mortality rates among C-DVT were significantly higher than in patients without. C-DVT was associated with reduced overall survival in patients with unsuspected PE (hazard ratio 1.33, 95% confidence interval 1.09–1.63, *p* = 0.005). In conclusion, C-DVT in cancer patients who present with unsuspected PE is common and is associated with an increased risk of VTE recurrence and poor short- and long-term survival. Identifying other venous thrombi in cancer patients presenting with unsuspected PE is recommended and can guide the management plan. For patients with isolated incidental subsegmental pulmonary embolism and concomitant deep vein thrombosis, initiating anticoagulants if no contraindications exist is recommended.

## 1. Introduction

Venous thromboembolism (VTE) is a common and well-known concerning complication linked to the existence of cancer (occult or overt) and its progression [1,2,3,4]. Cancer patients have a significantly higher risk of developing VTE during their disease course when compared with the general population [5,6,7,8] given that abnormalities in all three components of the Virchow triad (i.e., venous stasis, vessel wall injury, and hypercoagulability) are present in cancer patients [9,10,11,12]. In patients with malignancies, the clinical presentation of pulmonary embolism (PE) varies substantially, ranging from an asymptomatic or incidental finding to symptoms related to severe hemodynamic instability and even death [13]. More than 20–40% of cancer patients with acute PE have been shown to have concomitant deep vein thrombosis (DVT) at the time of diagnosis of the PE [13,14,15,16].

Remarkable advances in medical imaging studies, including computed tomography (CT), have allowed for improved visualization of the pulmonary arterial tree, which has led to an increase in the detection of PE as an incidental finding [17,18]. During routine staging or follow-up imaging studies, which are frequently ordered for cancer patients, incidental VTEs, including incidental PEs can be identified [17,19]. In the emergency department, despite the differences in the diagnostic approach, the management plan for cancer patients with an incidental or unsuspected PE are comparable to those used in suspected PE [20]. The inadequate evidence supporting the current clinical guidelines for the diagnostic and management plans in cancer patients with an incidental PE may contribute to a wide variation in practice patterns, in which additional diagnostic investigations (such as Doppler ultrasound or laboratory clotting biomarkers) and the use of anticoagulants are based on anecdotal or personal clinical experience or expertise of the involved clinicians. This has possibly created a practice heterogeneity amongst clinicians regarding the timing of anticoagulation, agent selection, and the duration of treatment for incidental VTE in cancer patients [21], despite the consistent recommendation statements in the current guidelines [22,23,24,25,26].

Determining the characteristics and clinical presentation of an incidental PE is important to establish the optimal diagnostic and management plan including imaging studies, follow-up, and anticoagulation. Several studies have already examined incidental or unsuspected PEs in cancer patients and identified the characteristics, management, and outcomes of PE in these patients [18,20,27,28,29,30]. In the current study, we sought to characterize concomitant incidental thrombosis (C-DVT) in cancer patients presenting with unsuspected PE and determine the potential impact of C-DVT on the patient’s short- and long-term survival and risk of VTE recurrence.

## 2. Materials and Methods

### 2.1. Patient Cohort, Data Collection, and Interobserver Agreement

To identify all consecutive cancer patients who visited our institution’s emergency department with unsuspected PE, we queried The University of Texas MD Anderson Cancer Center institutional billing databases for the period between 1 January 2006 and 1 January 2016, for the diagnosis of PE in patients who had undergone CT studies of the chest with intravenous contrast (excluding CT pulmonary angiogram or CT PE protocol which are usually ordered for suspected PEs) within 24 h before or during the emergency department visit. The records of the identified patients were manually reviewed by trained abstractors to confirm the presence of acute unsuspected PE. Radiology reports were used to confirm the presence of acute PE incidentally found on a chest CT study with contrast. Central PE was defined as acute PE within the main (including saddle embolism) and interlobar pulmonary arteries up to the lobar level. Advanced cancer stage was defined as stage IV for solid tumors based on the American Joint Committee on Cancer anatomic stage/prognostic groups, grade IV for brain and spinal cord tumors based on the World Health Organization groups, or hematologic malignancies in relapsed or refractory phases. An expert thoracic radiologist reviewed the images where reports stated questionable findings of PE (54 reports) and determined whether an acute incidental PE was present.

All imaging study reports (including abdominal and pelvic CT studies, extremity Doppler ultrasound ordered after the incidental PE was identified, CT study of the neck, and the same indexed chest CT study with contrast) for patients with confirmed unsuspected PE were reviewed to determine the presence or absence of C-DVT. C-DVT was defined as acute DVT reported on any imaging study carried out within 72 h (to assure the concomitant status) after the emergency department presentation. The institution’s electronic medical record system was used to collect demographic, clinical, and radiologic data for each patient. The institution pharmacy database was used to identify patients who had anticoagulants prescription prior to their emergency department visit. 

Exclusion criteria were as follows: (1) non-cancer patient, (2) absence of acute incidental PE (i.e., absence of true filling defects in the pulmonary arterial tree or pulmonary arterial filling defects were attributed to chronic PE or tumor thrombus), (3) clinically suspected PE (i.e., not incidental based on physician notes), (4) prior diagnosis of VTE within one month before incidental PE discovery, and (5) incomplete medical records. A data dictionary, collection form, and biweekly meetings were used to ensure accurate and uniform abstraction. A random sample of 46 final charts (5%) was reviewed by different abstractors to assess interrater agreement. This analysis yielded a kappa value of 0.843, implying almost perfect agreement among the abstractors. 

### 2.2. Data and Statistical Analysis 

Standard descriptive statistics were used to analyze and report patient demographics and clinical characteristics. The chi-square test was used to compare frequencies for the categorical data, and the nonparametric Wilcoxon-Mann-Whitney test was used to compare continuous variables because the normality assumption was not met for all continuous variables. Univariate logistic regression analysis was used to identify associations between clinical factors, including the presence of C-DVT and VTE recurrence within 6 months of presentation. Next, a multivariable logistic regression model that included all the statistically significant variables from the univariate analysis was constructed, reporting the odds ratio (OR) with 95% confidence interval (95% CI). For this analysis, the site of cancer was grouped based on their previously reported risk stratification for VTE: Very high risk (stomach, pancreas, primary brain tumor); high risk (lung, lymphoma, gynecologic, bladder, testicular, and renal tumors); and low risk (all other tumors) [22]. The 30-, and 90-day mortality rates were compared between patients with and without C-DVT. Univariate and multivariable Cox proportional hazards models were used to investigate the association between different clinical variables and survival for all patients, reporting the hazard ratio (HR) and 95% CI. To estimate the difference in overall survival between patients with or without C-DVT, we conducted a Kaplan-Meier survival analysis followed by a log-rank test. Events were censored at the last contact date or 2 years after presentation. A two-sided *p* < 0.05 was considered statistically significant.

All statistical analyses were carried out using R software version 4.0.2 (The R Foundation, http://www.r-project.org accessed on 29 May 2021). The Institutional Review Board of MD Anderson approved the study and granted a waiver of informed consent. Anonymized patient-level data that are compliant with Health Insurance Portability and Accountability Act regulations will be shared upon acquiring MD Anderson Institutional Review Board approval.

## 3. Results

### 3.1. Characteristics of Cancer Patients with Unsuspected PE with or without C-DVT

The final analysis included 904 eligible patients with unsuspected PE (Figure S1). Of these, 189 patients (20.9%) had C-DVT. Table 1 shows the common clinical characteristics of the final cohort. Most patients had advanced-stage cancer and were receiving active cancer treatment. Sixty-eight (7.5%) patients had anticoagulants prescription within 90 days before emergency department presentation. The median Charlson comorbidity index score for the whole cohort was 6 (interquartile range: 5–7) which was not significantly different between patients with or without C-DVT (*p* = 0.247). 

The top four sites for C-DVT were femoral (45.0%), popliteal (23.3%), iliac (22.8%), and inferior vena cava (9.0%), and most C-DVTs (56.6%) were found in CT imaging studies of the abdomen and pelvis (Table 2). The remaining C-DVTs were discovered on Doppler ultrasound that was ordered after the discovery of the PE (33.3%), a non-PE protocol CT study of the chest (9.5%), and CT study of the neck (2.1%). 

Most the patients (82.5%) with C-DVTs had no symptoms related to the DVT at the time of the DVT diagnosis (Table S1). Patients with a central unsuspected PE had a significantly higher incidence (*p* = 0.004) of C-DVT compared with patients with a peripheral unsuspected PE. The presence of C-DVT was confirmed in 28.7% of the patients with a saddle or main unsuspected PE, 19.8% of patients with an interlobar or lobar unsuspected PE, and 16.9% of patients with a segmental or subsegmental unsuspected PE (Table S2). Most patients were prescribed anticoagulants for the PE upon discharge (86.2% of patients with C-DVT and 92.4% of patients without C-DVT); however, an inferior vena cava filter was placed in 11.1% of patients with C-DVT compared with only 4.3% of patients without C-DVT (Table S3).

### 3.2. Increased Odds of VTE Recurrence within 6 Months in Cancer Patients with C-DVT

In this case, 71 patients had acute VTE recurrence within 6 months after presenting to the emergency department with unsuspected PE. Of these, 59 (83.1%) had DVT, 9 (12.7%) had PE, and 3 (4.2%) had both PE and DVT as their recurrent acute VTE events. The presence of C-DVT at initial presentation was associated with more than twice the odds of developing VTE recurrence within 6 months in both the univariate (OR 2.22, 95% CI 1.31–3.68, *p* = 0.002) and multivariable analyses (OR 2.07, 95% CI 1.21–3.48, *p* = 0.007), as shown in Table 3. Very high-risk and high-risk cancer sites were also main predictors of VTE recurrence when compared to the low-risk sites. The multivariable OR for the very high-risk sites (stomach, pancreas, primary brain tumor) was 2.30 (95% CI = 1.01–4.89, *p* = 0.037), while it was 2.11 (95% CI = 1.24–3.64, 0.006) for the high-risk cancer sites (lung, lymphoma, gynecologic, bladder, testicular, and renal tumors). Other predictors of VTE recurrence in the multivariable analysis were age (OR 0.97, 95% CI 0.95–0.99, *p* = 0.005) and advanced cancer stage (OR 2.41, 95% CI 0.96–8.09), *p* = 0.097).

Similar results were observed in a subgroup analysis that included only patients who were prescribed anticoagulants at discharge (Table S4); in this subgroup, patients with C-DVT had double the odds of developing VTE recurrence within 6 months after presentation (OR 2.27, 95% CI 1.27–3.96, *p* = 0.005). In another subgroup analysis, patients with peripheral unsuspected PE with C-DVT (Table S5) had almost 3 times the odds of developing VTE recurrence within 6 months after presentation (OR 2.59, 95% CI 1.29–5.05, *p* = 0.006).

### 3.3. Association of C-DVT with Poor Survival in Cancer Patients with Unsuspected PE

**Figure 1:**
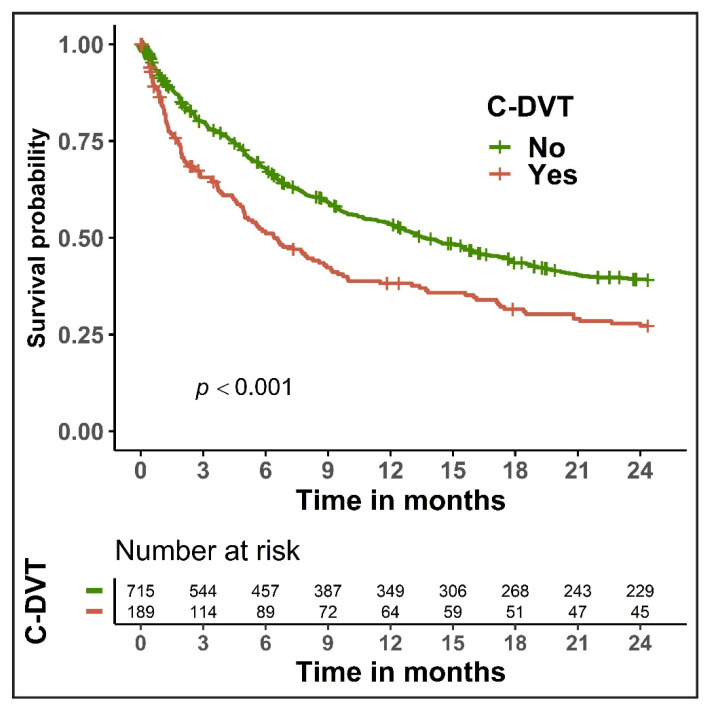
Kaplan-Meier curves for overall survival in cancer patients with unsuspected pulmonary embolism, stratified by the presence of concomitant deep vein thrombosis (C-DVT).

The 30-day and 90-day mortality rates among patients with C-DVT were 15.3% and 32.8%, respectively, which was significantly higher (*p* < 0.01 for both) than in cancer patients without C-DVT (8.5% and 19.4%, respectively; Table S6). The presence of C-DVT in cancer patients presenting with unsuspected PE was associated with poor overall survival (*p* < 0.001; Figure 1). In the univariate Cox regression analysis, C-DVT was associated with reduced overall survival (HR 1.49, 95% CI 1.22–1.82, *p* < 0.001). A similar effect (HR 1.33, 95% CI 1.09–1.63, *p* = 0.005) was observed in the multivariable analysis after controlling for cancer type, cancer stage, and Charlson comorbidity index (Table 4). In the multivariable analysis, certain cancer types (lung, gynecologic, gastrointestinal) and advanced cancer stage were also main significant predictors of survival in our cohort.

## 4. Discussion

Malignancy by itself is a main risk factor for the development of VTE, but cancer patients have multiple other clinical risk factors, including chemotherapy, multiple surgeries, and immobility [16,31,32,33,34]. Despite being unsuspected or incidentally discovered, incidental VTEs appear to be a substantial risk for cancer patients, and this may influence patient morbidity and mortality [14,27,30]. In the current study, we used data collected from cancer patients presenting to our comprehensive cancer center over a 10-year period to characterize C-DVT in cancer patients presenting with unsuspected PE, describing the outcomes of patients with these events. Of the patients who presented with unsuspected PE, 20.9% had C-DVT, indicating a high rate of occurrence. Patients with C-DVT had almost twice the odds of developing VTE recurrence compared with those without C-DVT (OR 2.07, 95% CI 1.21–3.48, *p* = 0.007). In this case, 30-day and 90-day mortality rates were also significantly higher among patients with C-DVT (15.3% and 32.8%, respectively) when compared with patients without C-DVT (8.5% and 19.4%, respectively; *p* < 0.01). C-DVT was also associated with reduced overall survival (HR 1.33, 95% CI 1.09–1.63, *p* = 0.005). 

In cancer patients with VTEs, an estimated 35–50% of the VTEs are discovered incidentally [35,36]. In contrast with suspected VTEs, for incidental or unsuspected VTEs, the evidence supporting the current guidelines in terms of approach, management, and outcomes is still inadequate [22,24,25,26,37,38]. The controversy mainly appears for peripheral isolated PEs, where the existence of true emboli in distal regions of the pulmonary arterial tree is weighed against a false-positive result [39,40]. Expert chest radiologists frequently rectify and report the absence of a PE after a false initial diagnosis by a junior or non-expert (not specialized in the chest) radiologist [39,40]. In cancer patients, a misdiagnosed PE could lead to catastrophic outcomes. Unnecessary anticoagulants increase the risk of major bleeding in cancer patients, who are known to have complex coagulopathy [41,42]. In addition, the patient will be flagged as having a history of PE, which may lead to frequent and maybe unnecessary imaging studies to investigate recurrence if the patient presents later with VTE-related symptoms [43]. Therefore, identifying other characteristics that may affect survival and are associated with increased risk of recurrence can guide emergency department physicians, hematologists, and oncologists in developing the proper diagnostic and management plan for cancer patients with incidental or unsuspected PE, specifically for isolated peripheral incidental PEs.

The American Society of Clinical Oncology guideline recommends treating incidental PEs much the same as symptomatic ones, except for isolated subsegmental PEs, which the guideline recommends treating on a case-by-case basis [22]. The most recent American Society of Hematology guidelines for the management of VTE suggests (although the evidence has very low certainty) short-term anticoagulation therapy for patients with incidental or unsuspected PE [24]. The same guideline recommends the use of clinical judgment in administering anticoagulants to a patient with subsegmental PE, either symptomatic or incidental [24]. CHEST 2021 guidelines recommended treating incidental PE in the same manner as symptomatic PE; however, this is considered a weak recommendation, with only moderate-certainty evidence [26]. The European Society of Cardiology 2019 guidelines for the diagnosis and management of acute PE support a treatment approach for cancer patients with incidental PE similar to that of patients with symptomatic PE, but only if the incidental PE involves “segmental or more proximal branches, multiple subsegmental vessels, or a single subsegmental vessel in association with proven DVT” [23], p. 579. Interestingly, no recommendation was given for the treatment of a single subsegmental PE in the absence of DVT [23]. In the current version of the Spanish Society of Medical Oncology clinical guideline for VTE and cancer, an individualized approach is recommended for patients with subsegmental PE; nevertheless, the guideline suggests considering anticoagulation therapy, with a 2C level of evidence [25]. In addition to these guidelines, various observational studies have investigated the proper management of incidental PE, but there is little to indicate a consensus about whether all incidental PEs should be treated such as symptomatic PEs because of the same rate of recurrence [20,44], or whether an individualized approach for patients with subsegmental PE is more proper [28].

Various studies for suspected PE have shown that concomitant DVT alters short-term mortality, and its presence can be used to improve risk stratification in patients with intermediate to low risk for short-term complications from PE [45,46,47,48]. A recent study by Barca-Hernando et al. that included 200 cancer patients with incidental PE, of which 62 patients (31.0%) had C-DVT, concluded that the presence of C-DVT was not associated with poorer survival (univariate analysis: HR 1.09, 95% CI 0.43–2.75, *p* = 0.855) [14]. However, the small sample size of that study may have limited the statistical power to detect a difference in survival outcomes, and the study did not include a multivariable analysis for C-DVT, so other factors that influence survival could have masked the true impact of C-DVT on survival. In the current study, which had a large enough sample size to support a multivariable analysis, the results indicate an association between the presence of C-DVT in cancer patients with incidental PE and poor short- and long-term survival outcomes. 

We also found that the odds of VTE recurrence within 6 months of PE presentation were more than two times higher in patients with C-DVT. To the best of our knowledge, ours is the first study to show such an association. Given the lack of firm evidence or guidelines on the proper management of incidental PEs [22,24,25,26,37,38], the presence of C-DVT can be one of the main factors favoring the prescription of anticoagulants, especially in patients with a single peripheral PE, including isolated subsegmental PEs. However, the cost-effectiveness of searching for C-DVT in cancer patients with a single peripheral PE along with other considerations including the risk on the patients and utilization of resource needs to be investigated. In addition, cancer type is a known important factor that influences the risk of VTE [16,22,32]. While there was no significant difference in our cohort in the type of cancer between patients with or without C-DVT, cancer types stratified by their VTE risk were significant predictors of VTE recurrence. High-risk cancer types including gastric, pancreatic, and ovarian that are known to have an increased risk of thrombosis need to be focused on in the management and follow-up plans, especially in patients with advanced and active cancer.

For the therapeutic plan in these patients, therapeutic anticoagulants are recommended by the current guidelines regardless of the presence of symptoms related to VTE [22,24,49]. These guidelines strongly suggest using the same initial short- and long-term management strategies for most patients with incidental PE as those used in patients with symptomatic PE [22,24,49], except for in patients with isolated incidental subsegmental PE, which some guidelines recommend treating based on clinical judgment. For these patients with isolated incidental subsegmental PE, the presence of C-DVT strongly suggests an increased risk of VTE recurrence, and therefore initiating anticoagulants if no contraindications exist is recommended. Assessment for bleeding risk is critical in cancer patients owing to the complexity of related factors, and the risk is amplified in patients with thrombocytopenia, active cancer therapy, and widespread metastasis, as well as in certain high-risk cancer types, including primary luminal gastrointestinal cancer, genitourinary cancer, metastatic melanoma, and renal tumors. For patients with low bleeding risk, direct oral anticoagulants, including apixaban, edoxaban, and rivaroxaban, are preferred. For patients with high bleeding risk, including patients with gastroesophageal and gastric cancer, low molecular weight heparin, including dalteparin and enoxaparin, is preferred. Other options, including vitamin K antagonists, fondaparinux, or unfractionated heparin, can be considered based on the appropriateness of the case and the availability of the medications [22,24,50]. In patients for whom anticoagulants are contraindicated, an inferior vena cava filter should be considered, with close follow-up and consideration for anticoagulants if the contraindication is resolved [50,51,52]. 

The main limitations of the current study are related to its retrospective nature. Particularly important is the true incidence of C-DVT in cancer patients with unsuspected PE, which could be different than the 20.9% reported in the current study. In this retrospective study, identifying C-DVT events depended highly on the types of imaging studies carried out around the time of the incidental PE discovery, and thus our reported rate can be an underestimation. This limitation can be overcome only by a prospective study in which imaging studies to detect other thromboses are supported by funding for the study if not already carried out in the course of standard clinical care. Additionally, although we reported whether patients were discharged with anticoagulants, the rate of compliance and duration of treatment could not be effectively assessed retrospectively. Similarly, for patients identified to have anticoagulants prescription within 90 days prior to presentation, an important factor that could have influenced the risk of VTE, nonadherence to anticoagulants, and if some patients had prescriptions from outside our institution could not be assessed due to the nature of this study. However, for patients who could be safely given anticoagulants upon discharge, we expect a similar or longer duration of treatment for patients with C-DVT compared with those without C-DVT, which further supports our VTE recurrence analysis. Another limitation of the current study is identifying the incidence of recurrent VTE, which could have been detected and treated outside our institution and thus not included in our dataset. To minimize the impact of this limitation, we reviewed the follow-up physician notes to look for VTE events diagnosed and treated outside our institution, along with all imaging studies carried out at MD Anderson to check whether any VTE occurred outside our institution. 

## 5. Conclusions

In summary, C-DVT in cancer patients presenting with unsuspected PE is common, and C-DVT is associated with poor short- and long-term outcomes in these patients. The 30- and 90-day mortality rates among patients with C-DVT are significantly higher than those for cancer patients without C-DVT. In addition, our results showed that the presence of C-DVT is a predictor of VTE recurrence, resulting in twice the odds of developing VTE recurrence within 6 months from the initial presentation. Proper identification of C-DVT in cancer patients presenting with incidental or unsuspected PE, especially in patients with peripheral PE, is critical and can help guide anticoagulation management and oncology care to improve patient outcomes. Initiating anticoagulants if no contraindications exist is recommended for patients with isolated incidental subsegmental PE in the presence of C-DVT.

## Figures and Tables

**Table 1:** Demographic and clinical characteristics of cancer patients with incidental pulmonary embolism with or without concomitant incidental thrombosis (*n* = 904).

Characteristic	Concomitant Incidental Thrombosis, no. (%)		*p*
	No	Yes	
**Total**	715 (79.1)	189 (20.9)	
**Median age (IQR), years**	63 (55–70)	63 (53–70)	0.870
**Sex**			0.146
Female	317 (44.3)	95 (50.3)	
Male	398 (55.7)	94 (49.7)	
**Race**			0.604
Non-White	164 (22.9)	40 (21.2)	
White	551 (77.1)	149 (78.8)	
**Cancer type**			0.188
Lung	115 (16.1)	24 (12.7)	
Colorectal	78 (10.9)	26 (13.8)	
Breast	63 (8.8)	6 (3.2)	
Lymphoma	57 (8.0)	13 (6.9)	
Urinary	51 (7.1)	17 (9.0)	
Female genital	49 (6.9)	14 (7.4)	
Sarcoma	47 (6.6)	9 (4.8)	
Gastroesophageal	44 (6.2)	13 (6.9)	
Pancreas	41 (5.7)	14 (7.4)	
Melanoma	39 (5.5)	16 (8.5)	
Other gastrointestinal	36 (5.0)	14 (7.4)	
Others	95 (13.3)	23 (12.2)	
**Cancer stage**			0.882
Local	57 (8.0)	13 (6.9)	
Advanced	576 (80.6)	154 (81.5)	
Hematologic	82 (11.5)	22 (11.6)	
**Active cancer therapy**			0.966
No	319 (44.6)	84 (44.4)	
Yes	396 (55.4)	105 (55.6)	
**Anticoagulants prescription within 90 days before ED presentation**			0.073
No	667 (93.3)	169 (89.4)	
Yes	48 (6.7)	20 (10.6)	
**Median CCI (IQR)**	6 (4–7)	6 (6–7)	0.247

Abbreviations: IQR, interquartile range; ED, emergency department; CCI, Charlson comorbidity index.

**Table 2:** Characteristics of concomitant deep vein thrombosis (C-DVT) in cancer patients with unsuspected pulmonary embolism (*n* = 189).

Characteristic	No. of Patients (%)
C-DVT location *	
Femoral	85 (45.0)
Popliteal	44 (23.3)
Iliac	43 (22.8)
Inferior vena cava	17 (9.0)
Subclavian	12 (6.3)
Jugular	12 (6.3)
Saphenous	10 (5.3)
Superior vena cava	9 (4.8)
Brachiocephalic	8 (4.2)
Axillary	6 (3.2)
Basilic	5 (2.6)
Others	21 (11.1)
**Imaging study type**	
CT of the abdomen and pelvis	104 (55.0)
Doppler ultrasound	63 (33.3)
CT of the chest	18 (9.5)
CT of the neck	4 (2.1)

Abbreviations: CT, computed tomography. * Some patients had more than one C-DVT.

**Table 3:** Univariate and multivariable logistic regression analyses of clinical factors associated with venous thromboembolism recurrence within 6 months in cancer patients with unsuspected pulmonary embolism (*n* = 904).

Variable	Univariate		Multivariable	
	OR (95% CI)	*p*	OR (95% CI)	*p*
**Age, years**	0.97 (0.96–0.99)	0.008	0.97 (0.95–0.99)	0.005
**CCI**	0.94 (0.84–1.05)	0.235	-	-
Site of cancer *				
Low risk	Reference			
High risk	1.91 (1.13–3.25)	0.016	2.11 (1.24–3.64)	0.006
Very high risk	2.39 (1.06–5.02)	0.026	2.30 (1.01–4.89)	0.037
**Cancer stage**				
Local	Reference			
Advanced	2.96 (1.2–9.85)	0.038	2.41 (0.96–8.09)	0.097
**Time from cancer diagnosis, months**	1.00 (0.99–1.01)	0.809	-	-
**C-DVT**				
No	Reference			
Yes	2.22 (1.31–3.68)	0.002	2.07 (1.21–3.48)	0.007

Abbreviations: OR, odds ratio; CI, confidence interval; CCI, Charlson comorbidity index; C-DVT, concomitant deep vein thrombosis. * Site of cancer grouping based on VTE risk: Very high risk (stomach, pancreas, primary brain tumor); high risk (lung, lymphoma, gynecologic, bladder, testicular, and renal tumors); low risk (all other tumors).

**Table 4:** Univariate and multivariable Cox proportional hazards analyses of overall survival in cancer patients with unsuspected pulmonary embolism (*n* = 904).

Variable	Univariate		Multivariable	
	HR (95% CI)	*p*	HR (95% CI)	*p*
**Age**	1.00 (0.99–1.19)	0.281	-	-
**Sex**				
Female	Reference			
Male	0.88 (0.74–1.04)	0.128	-	-
**Race**				
Non-White	Reference			
White	0.95 (0.77–1.17)	0.637	-	-
**CCI**	1.14 (1.09–1.18)	<0.001	1.11 (1.06–1.16)	<0.001
**Cancer type**				
Breast	Reference			
Gynecologic	2.25 (1.42–3.57)	<0.001	2.01 (1.27–3.20)	0.003
Gastrointestinal	1.97 (1.34–2.91)	<0.001	1.82 (1.23–2.69)	0.003
Lung	1.89 (1.25–2.86)	0.002	1.80 (1.19–2.72)	0.005
Lymphoma	0.77 (0.46–1.30)	0.330	0.96 (0.56–1.64)	0.882
Urinary	1.16 (0.72–1.88)	0.546	1.04 (0.64–1.68)	0.888
Other cancer types	1.44 (0.97–2.14)	0.073	1.27 (0.85–1.89)	0.243
**Cancer stage**				
Local	Reference			
Advanced	4.01 (2.84–5.65)	<0.001	3.68 (2.61–5.20)	<0.001
**C-DVT**				
No	Reference			
Yes	1.49 (1.22–1.82)	<0.001	1.33 (1.09–1.63)	0.005

Abbreviations: HR, hazard ratio; CI, confidence interval; CCI, Charlson comorbidity index; C-DVT, concomitant deep vein thrombosis.

## Data Availability

The data presented in this study are available on request from the corresponding author. The data are not publicly available and anonymized patient-level data that are compliant with Health Insurance Portability and Accountability Act regulations will be shared upon acquiring MD Anderson Institutional Review Board approval.

## Supplementary Materials

The following supporting information can be downloaded at: https://www.mdpi.com/article/10.3390/cancers14184510/s1, Figure S1: Flow chart of study eligibility; Table S1: Symptoms’ characteristics related to concomitant deep vein thrombosis in cancer patients with unsuspected pulmonary embolism at the time of deep vein thrombosis diagnosis; Table S2: Presence of concomitant deep vein thrombosis in cancer patients with an unsuspected pulmonary embolism (PE), stratified by location of the incidental PE (*n* = 904); Table S3: Management for cancer patients with unsuspected/incidental pulmonary embolism (*n* = 904) at discharge, stratified by the presence of concomitant deep vein thrombosis; Table S4: Univariate and multivariable logistic regression analyses of clinical factors associated with venous thromboembolism recurrence within 6 months in cancer patients with unsuspected pulmonary embolism who were discharged with anticoagulants (*n* = 824); Table S5: Univariate and multivariable logistic regression analyses of clinical factors associated with venous thromboembolism recurrence within 6 months in cancer patients with peripheral unsuspected pulmonary embolism; Table S6: Short-term mortality rates for cancer patients with unsuspected pulmonary embolism with or without concomitant deep vein thrombosis (*n* = 904).
