# Supplementary material for: Concomitant Deep Vein Thrombosis in Cancer Patients with Unsuspected Pulmonary Embolism
Source: Cancers (Basel). 2022 Sep 17;14(18):4510. doi: 10.3390/cancers14184510 (PMC9496711; doi:10.3390/cancers14184510)
Supplement: Supplementary file 1 [file cancers-14-04510-s001.zip › cancers-1889904-supplementary.pdf]

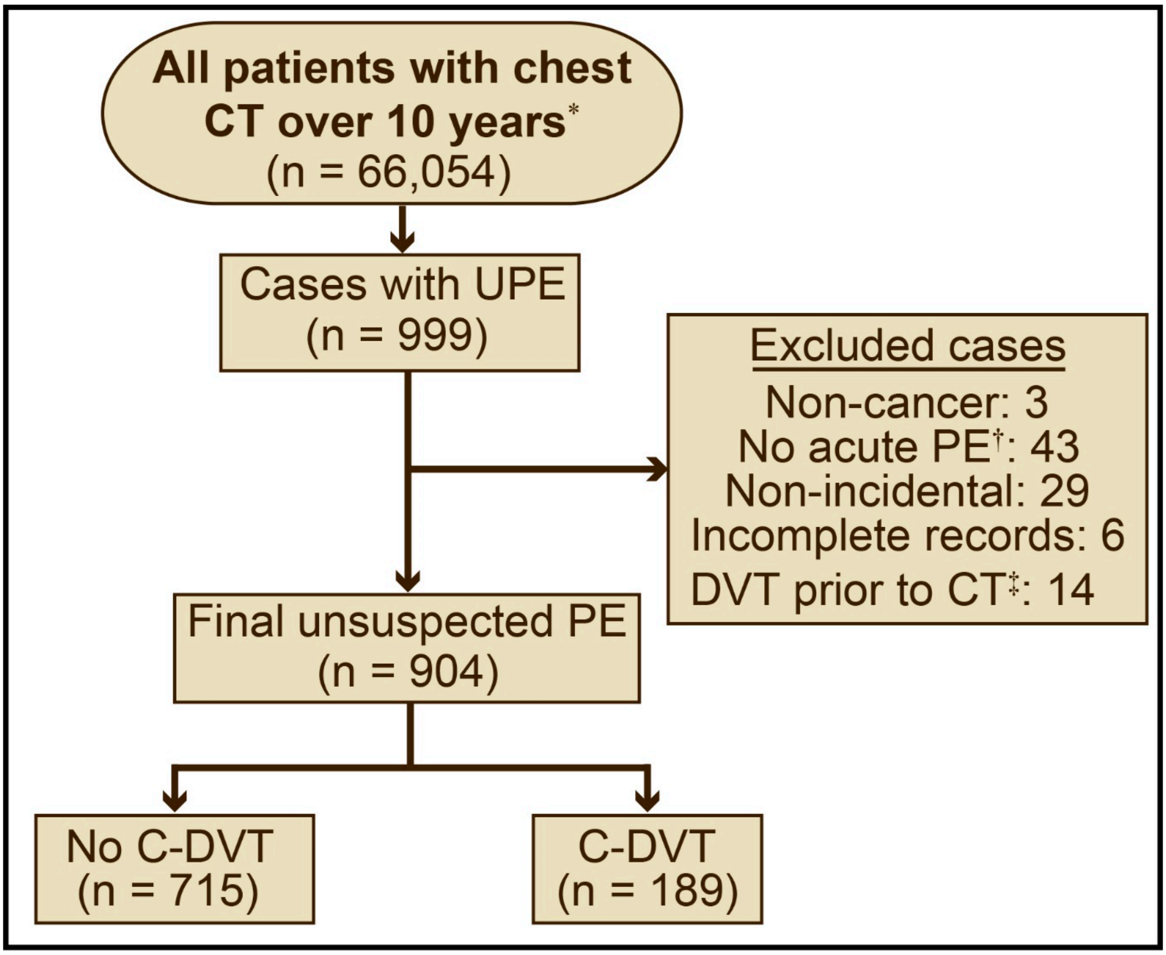

Figure S1. Flow chart of study eligibility. Abbreviations: UPE, unsuspected or incidental pulmonary embolisms; DVT, deep vein thrombosis; CT, computed tomography; C-DVT, concomitant deep vein thrombosis. * *CT chest with intravenous contrast excluding CT pulmonary angiogram or CT PE protocol which are usually ordered for suspected PEs †Indicates absence of acute incidental PE (i.e., absence of true filling defects in the pulmonary arterial tree or pulmonary arterial filling defects were attributed to chronic PE or tumor thrombus). ‡Indicates prior diagnosis of DVT within one month before incidental PE discovery.*

Table S1. Symptoms' characteristics related to concomitant deep vein thrombosis in cancer patients with unsuspected pulmonary embolism at the time of deep vein thrombosis diagnosis

Symptoms	No. of patients (%)
None (asymptomatic)	156 (82.5)
Extremity swelling	23 (12.2)
Extremity pain and swelling	5 (2.6)
Extremity pain	5 (2.6)

Table S2. Presence of concomitant deep vein thrombosis in cancer patients with an unsuspected pulmonary embolism (PE), stratified by location of the incidental PE (n = 904).

	Concomitant deep vein thrombosis			
**Incidental PE location**	**No**	**Yes**	**Percentage**	***P***
Subsegmental/segmental	261	53	16.9	
Lobar/interlobar	300	74	19.8	0.004
Main/saddle	154	62	28.7	

Table S3. Management for cancer patients with unsuspected/incidental pulmonary embolism (n = 904) at discharge, stratified by the presence of concomitant deep vein thrombosis.

	Concomitant deep vein thrombosis		
**Treatment**	**No**	**Yes**	***P***
Anticoagulants	661 (92.4)	163 (86.2)	
IVC filter only	31 (4.3)	21 (11.1)	0.002
None	23 (3.2)	5 (2.6)	

Abbreviations: IVC, inferior vena cava.

Table S4. Univariate and multivariable logistic regression analyses of clinical factors associated with venous thromboembolism recurrence within 6 months in cancer patients with unsuspected pulmonary embolism who were discharged with anticoagulants (n = 824).

	**Univariate**		**Multivariable**	
**Variable**	**OR (95% CI)**	***P***	**OR (95% CI)**	***P***
Age	0.98 (0.96-1.00)	**0.024**	0.97 (0.95-0.99)	**0.007**
CCI	0.91 (0.81-1.02)	0.114	-	-
Site of cancer*				
Low risk		Reference		
High risk	2.21 (1.27-3.93)	**0.006**	2.49 (1.41-4.48)	**0.002**
Very high risk	2.52 (1.02-5.71)	**0.033**	2.54 (1.01-5.84)	**0.034**
Cancer stage				
Local		Reference		
Advanced	2.71 (1.09-9.06)	0.058	-	-
Time from cancer diagnosis	1.00 (0.99-1.01)	0.964	-	-
C-DVT				
No		Reference		
Yes	2.24 (1.26-3.86)	**0.005**	2.27 (1.27-3.96)	**0.005**

Abbreviations: OR, odds ratio; CI, confidence interval; CCI, Charlson comorbidity index, C-DVT; concomitant deep vein thrombosis.

* Site of cancer grouping based on VTE risk: Very high risk (stomach, pancreas, primary brain tumor); high risk (lung, lymphoma, gynecologic, bladder, testicular, and renal tumors); low risk (all other tumors).

Table S5. Univariate and multivariable logistic regression analyses of clinical factors associated with venous thromboembolism recurrence within 6 months in cancer patients with peripheral unsuspected pulmonary embolism.

	**Univariate**		**Multivariable**	
**Variable**	**OR (95% CI)**	***P***	**OR (95% CI)**	***P***
Age	0.97 (0.95-0.99)	**0.009**	0.97 (0.94-0.99)	**0.005**
CCI	1.01 (0.88-1.17)	0.882	-	-
Site of cancer*				
Low risk			Reference	
High risk	1.79 (0.91-3.57)	0.094	1.98 (0.99-4.01)	0.053
Very high risk	2.18 (0.76-5.56)	0.118	2.11 (0.72-5.52)	0.144
Cancer stage				
Local			Reference	
Advanced	2.43 (0.86-10.18)	0.145	-	-
Time from cancer diagnosis	1.00 (0.98-1.01)	0.500	-	-
C-DVT				
No			Reference	
Yes	2.67 (1.34-5.13)	**0.004**	2.59 (1.29-5.05)	**0.006**

Abbreviations: OR, odds ratio; CI, confidence interval; CCI, Charlson comorbidity index; C-DVT; concomitant deep vein thrombosis.

* Site of cancer grouping based on VTE risk: Very high risk (stomach, pancreas, primary brain tumor); high risk (lung, lymphoma, gynecologic, bladder, testicular, and renal tumors); low risk (all other tumors).

Table S6. Short-term mortality rates for cancer patients with unsuspected pulmonary embolism with or without concomitant deep vein thrombosis (n = 904).

	**Concomitant deep vein thrombosis, no. (%)**		
**Mortality**	**Yes, n=189**	**No, n=715**	***P***
15 days	15 (7.9)	31 (4.3)	**0.045**
30 days	29 (15.3)	61 (8.5)	**0.005**
90 days	62 (32.8)	139 (19.4)	**<0.001**
